# Dual-Stream Difference Modeling with Deep-Guided Multiscale Fusion for Mangrove Change Detection

**DOI:** 10.3390/s26051701

**Published:** 2026-03-08

**Authors:** Xin Wang, Shuai Tang, Qin Qin, Shunqi Yuan, Xiansheng Liang

**Affiliations:** 1 School of Computer Science and Information Security, Guilin University of Electronic Technology, Guilin 541004, China; 2 School of Computer Engineering, Guilin University of Electronic Technology, Beihai 536000, China; 3 School of Electronic Information, Guilin University of Electronic Technology, Beihai 536000, China

**Keywords:** change detection, mangrove, remote sensing, feature fusion, semantic guidance

## Abstract

Accurate mangrove change detection is important for coastal ecosystem monitoring but remains challenging due to tidal disturbances, unstable land–water boundaries, and multi-scale distribution variability. Tidal fluctuations introduce spectral variations that obscure real changes. As a result, existing deep learning methods face difficulties in distinguishing tide-induced pseudo-changes while balancing semantic consistency and boundary accuracy. To address these issues, we propose DSDGMNet, which incorporates Dual-Stream Difference Modeling and Deep-Guided Multiscale Fusion. The dual-stream difference-driven strategy is designed to reduce tidal interference and improve sensitivity to true structural changes, and the deep-guided multiscale fusion module integrates global context with fine boundary details. Experiments on the GBCNR dataset show that DSDGMNet achieves an F1-score of 71.36% compared to 68.87% by SNUNet (Siamese Densely Connected UNet) and 66.39% by ChangeFormer. On the WHU-CD dataset, DSDGMNet yields an F1-score of 91.38%, in comparison with 89.85% for DDLNet and 88.82% for ChangeFormer. These results suggest the method’s effectiveness for mangrove change detection in complex intertidal environments.

## 1. Introduction

Mangrove ecosystems serve as critical transitional zones linking land and sea, playing vital roles in maintaining coastal stability, conserving biodiversity, sequestering carbon, and mitigating climate change [1]. However, driven by multiple factors such as urban expansion, coastal development, aquaculture, and extreme weather events, mangroves worldwide face ongoing degradation with increasing ecological pressures. Therefore, timely and accurate monitoring of mangrove distribution and dynamic changes holds significant practical value for ecological conservation, environmental assessment, and resource management. Remote sensing technology, with its advantages of large-scale coverage, periodicity, and objectivity, is widely applied in mangrove monitoring, providing a reliable data foundation for revealing their spatiotemporal evolution [2].

With the advent of deep learning [3,4,5], deep neural networks have become the mainstream approach for remote sensing change detection, enabling more powerful feature learning in complex scenarios. Recent studies have advanced change detection in complex wetland environments, providing important insights for this work. Pan et al. proposed a spatio-temporal attention network (STANet) and a semantic change detection (SCD) framework to capture fine-grained “from-to” change information in wetlands [6,7]. Qian et al. addressed spectral similarity and pseudo-change in tidal wetlands with the Time-Spectral-Semantic Aware Convolutional Transformer (TSSA-CTNet) [8], integrating spectral and semantic priors to improve robustness across diverse wetland habitats. These studies highlight the importance of integrating spatio-temporal, spectral, and semantic information while suppressing environmental noise in wetland change detection. In addition, Luo et al. proposed the Bayesian Tile Attention Network (BTCDNet) [9] to alleviate data scarcity in mangrove mapping, introducing Bayesian prior guidance and efficient tile-based attention for robust and lightweight detection under limited sample.

Despite significant progress, most existing studies either focus on long-term sequence analysis or treat mangroves as a generic class within tidal wetlands, without explicitly addressing their unique identification challenges. Meanwhile, substantial advances have been made in the fundamental techniques underpinning our approach, particularly in bi-temporal feature fusion and multiscale representation learning, which provide a solid methodological foundation for this work.

For bi-temporal feature fusion, early Siamese-based approaches, such as FC-Siam (Fully Convolutional Siamese Networks) [10], employ symmetric encoders and simple fusion operations (e.g., differencing or concatenation), forming a classical paradigm for change detection. However, such linear and static fusion mechanisms are limited in modeling complex spatio-temporal interactions. Subsequent studies have enhanced feature interactions through dense connections [11,12], attention mechanisms [13], and adaptive gating [14], while recent works explored fine-grained pattern-aware fusion strategies, such as patch-based multi-head fusion in SRC-Net [15].

For multi-scale feature fusion, typical fusion strategies include upsampling, concatenation, and summing by elements [16,17,18]. However, early decoders mainly relied on linear fusion schemes and did not explicitly model cross-scale feature relationships, which limited their ability to achieve semantic consistency and boundary accuracy in ecohydrological coupling scenarios such as mangrove intertidal zones. Recent studies have proposed improved multiscale decoders. ChangFormer [19] and MFIN [20] adopt MLP-based fusion to enhance cross-scale integration, while MSFF-CDNet [21] introduces mask-guided fusion to capture gradual changes. ACMFNet [22] densely aggregates multi-level features to preserve spatial details.

Despite significant progress in related research, these methods still have shortcomings when directly applied to mangrove change detection. The challenges stem from two intertwined phenomena: the conflict between tidal-induced visual changes and structural stability, and the inherent scale inconsistency of mangrove boundaries.

**Figure 1:**
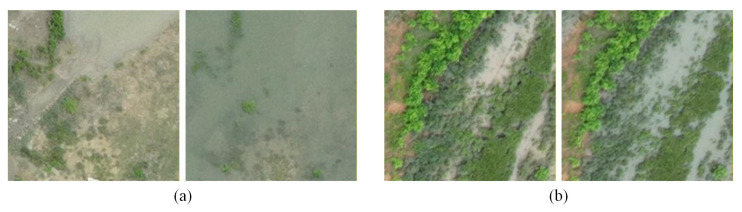
Example Images of Mangrove Changes. (**a**) Visual changes in water extent under tidal disturbance vs. ecological stability of mangroves.; (**b**) Multi-scale boundary drift among mangroves, water, and mudflats.

First, under tidal disturbance, the visual appearance of mangroves changes dramatically even when the ecosystem itself remains intact. As illustrated in Figure 1a, at different tidal heights, the extent of water bodies and boundary morphology undergo substantial shifts—yet the main mangrove structure shows no ecological change. This “apparent dramatic change vs. structural stability” conflict causes conventional methods to respond to tidal-induced pseudo-variations rather than genuine ecological changes.

Second, mangrove changes exhibit inherent scale inconsistency. At coarse scales, the overall distribution of mangroves may shift; at fine scales, boundaries between mangroves, water bodies, and mudflats fluctuate significantly with the tides (Figure 1b). This multi-scale boundary drift leads to blurred boundaries and easily submerged details, making it difficult to stabilize change detection across scales.

To address the aforementioned challenges, this paper employs a dual-level design approach encompassing Bi-Temporal Feature Fusion and cross-scale fusion. The primary contributions are as follows:

(1) To mitigate the issue where pronounced surface variations caused by differing tidal heights obscure the stable structure of mangroves, we propose the Dual-Stream Change Fusion (DSCF) module. This module achieves fine-grained alignment and fusion of dual-phase mangrove features through a difference-guided feature interaction mechanism.
(2) To address inconsistent mangrove change patterns across scales and unstable boundaries under tidal influence, we introduce the Deep-Guided Multiscale Decoder (DGMD). This module generates high-level semantic guidance information to adaptively weight and fuse multiscale features, thereby enhancing change boundary localization accuracy while preserving semantic consistency.

Compared to existing Siamese-based, attention-guided, and Transformer-based change detection methods, DSDGMNet exhibits two key distinctions. First, we explicitly model cross-temporal discrepancies through a dual-stream difference-driven interaction mechanism, which helps suppress tide-induced pseudo-changes. Second, we introduce a deep-guided multiscale fusion strategy that leverages high-level semantic representations to guide feature integration, aiming to improve semantic consistency and boundary precision. These design choices provide an alternative to conventional static or multiscale fusion strategies and, as demonstrated in our experiments, contribute to improved performance in mangrove change detection tasks.

## 2. Methods

### 2.1. Architecture Overview

**Figure 2:**
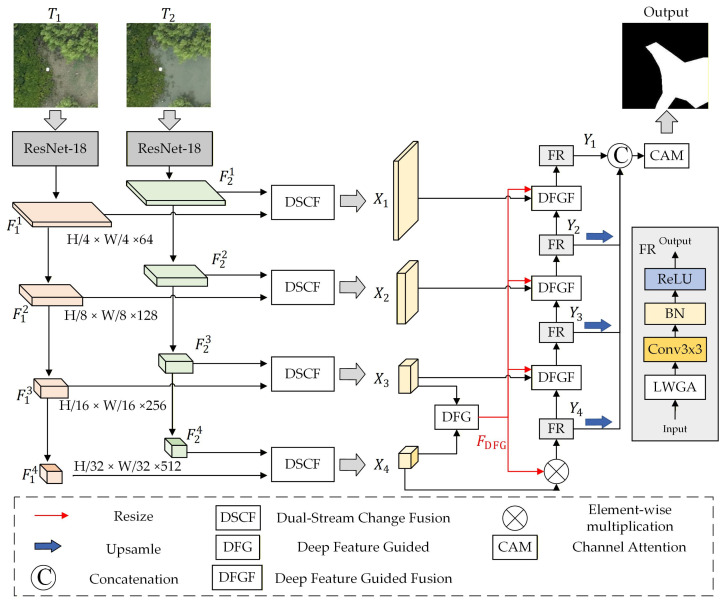
Overall architecture of the proposed DSDGMNet.

The overall architecture of the proposed network is illustrated in Figure 2, comprising two main components: the Bi-Temporal Feature Fusion encoder and the Multiscale Feature Fusion decoder. The core of the former is the proposed Dual-Stream Change Fusion (DSCF) module, which conducts difference-guided fine-grained alignment and fusion of bi-temporal features extracted by ResNet at various scales, thereby mitigating the dominance of non-structural differences over change information. The latter component is the Deep-Guided Multiscale Decoder (DGMD). It utilizes deep semantic features to generate guidance information, selectively adjusting multiscale features affected by tidal disturbances to facilitate a stable representation of mangrove change. This design addresses the challenges of coordinating multi-scale features and managing unstable change boundaries in mangrove imagery.

Specifically, the workflow of our proposed DSDGMNet is as follows. Given bi-temporal images $T_{1},T_{2}\in R^{3\times H\times W}$, a shared-weight ResNet-18 backbone extracts four levels of feature maps: $F_{j}^{i}$ for i=1…4, with spatial resolutions of H/4×W/4, H/8×W/8, H/16×W/16, and H/32×W/32, and channel dimensions of 64, 128, 256, and 512, respectively. For each level i, the bi-temporal feature pair $\left(F_{1}^{i},F_{2}^{i}\right)$ is fused by the Dual-Stream Change Fusion (DSCF) module, producing $X_{i}$. The deepest features $X_{3}$ and $X_{4}$ then pass through a Difference Feature Guidance (DFG) module to generate a guidance map $F_{DFG}$, which is multiplied with $X_{4}$ to enhance change-salient regions. A Feature Refinement (FR) module with Light Weight Grouped Attention (LWGA) module [23] further refines the result to produce $Y_{4}$. Subsequently, a cascade of DFGF modules performs cross-scale guided fusion from deep to shallow layers. Each DFGF module takes $X_{i}$, $Y_{i+1}$, and $F_{DFG}$ as inputs, producing refined features $Y_{3},Y_{2},Y_{1}$ sequentially. All refined features are summed and passed to a lightweight decoder [24] to generate the final change map. Detailed module descriptions are provide in the subsequent subsections.

Table 1 provides a detailed summary of the proposed architecture, specifying the input/output feature maps, channel dimensions, and spatial resolutions of each module, which is intended to improve the reproducibility and implementation clarity of our method.

### 2.2. Dual-Stream Change Fusion

Patch-Mode joint fusion in SRC-Net [15] is a representative approach for bi-temporal feature fusion, and the comparison results are shown in Table 2. where bi-temporal features are treated as time-varying signals and change modes are inferred probabilistically. In contrast, the proposed DSCF module adopts a difference-guided explicit modeling strategy. Specifically, we compute the difference map between bi-temporal features and use it as an attention prior to guide feature enhancement, aiming to mitigate tidal-induced pseudo-changes. This explicit modeling of change information, combined with the dual-branch design, contributes to more effective suppression of false changes in mangrove environments.

**Figure 3:**
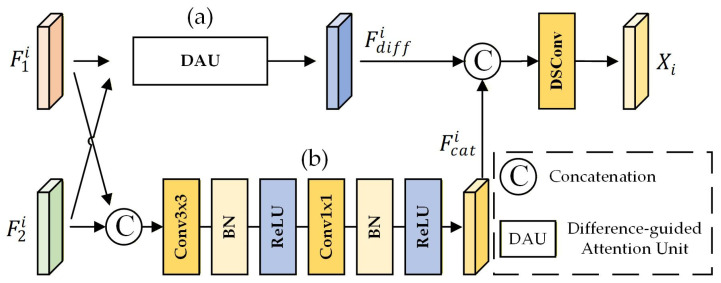
The architecture of the DSCF. (**a**) The difference branch; (**b**) The connection branch.

This module employs a difference-guided approach to achieve more robust interaction between bi-temporal features. It primarily consists of two branches: the difference branch (as shown in Figure 3a) and the connection branch (as shown in Figure 3b). Input features are $F_{1}^{i},\;F_{2\;}^{i}\;\in R^{C^{\prime }\times H^{\prime }\times W^{\prime }}$.

The connection branch maintains the overall structure and contextual information of mangroves by concatenating and convolving the bi-temporal features, thereby mitigating potential detail loss and noise amplification associated with pure difference calculations. It consists of a 3 × 3 convolutional block followed by a 1 × 1 convolutional block. The 3 × 3 block captures local spatial context, while the 1 × 1 block reduces the channel dimension to match the output of the subtraction branch. Each convolutional block comprises a convolution layer, batch normalization (BN), and a ReLU activation function. The input bi-temporal features are first concatenated along the channel dimension and then processed by this branch to yield the connection branch output, denoted as $F_{cat}\in R^{C^{\prime }\times H^{\prime }\times W^{\prime }}$. The difference branch employs a Difference-Aided Attention Unit (DAU), which explicitly models difference information derived from the bi-temporal features. It generates difference weights to modulate the original features, establishing a “difference-guided attention” mechanism for feature enhancement. This design enables the module to adaptively highlight regions of genuine change at both the channel and spatial levels while mitigating false change responses induced by factors such as tidal fluctuations and water coverage variations.

The bi-temporal features $F_{1}^{i}$ and $F_{2}^{i}$ are processed by the connection branch to generate $F_{cat}^{i}$, while $F_{diff}^{i}\in R^{C^{\prime }\times H^{\prime }\times W^{\prime }}$ is derived via the difference branch. Subsequently, these features are concatenated and processed through a single dilated convolution to yield the preliminary fusion result $X_{i}\in R^{C^{\prime }\times H^{\prime }\times W^{\prime }}$. This integration of the original difference features ensures that the dilated convolution incorporates both the refined context from the connection branch and the direct change signals from the difference branch, facilitating the adaptive integration of complementary information beyond mere feature stacking. By leveraging these complementary features, the two branches contribute to the alignment of bi-temporal mangrove features under complex tidal interference, thereby supporting the model’s capacity to discern genuine ecological changes.

#### Difference-Guided Attention Unit (DAU)

**Figure 4:**
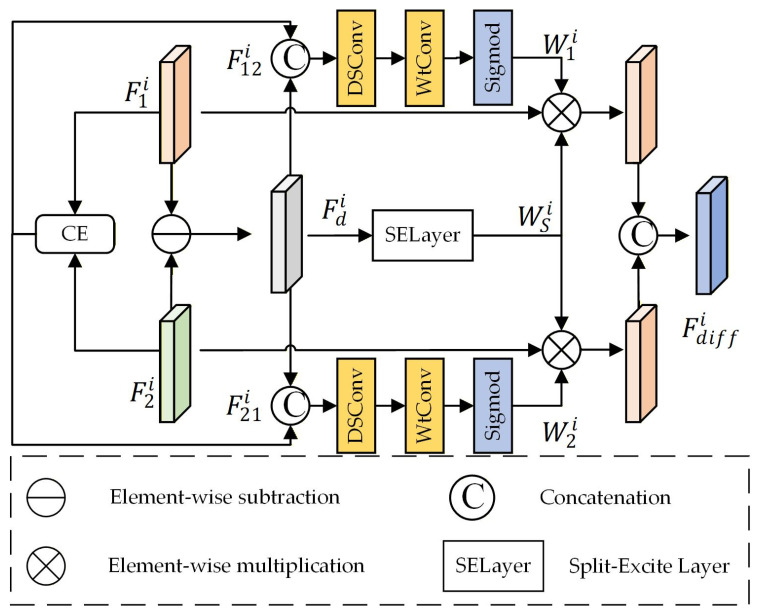
The architecture of the DAU.

The DAU serves as the core of the difference branch, with its architecture illustrated in Figure 4. The module first computes the difference map between the two input feature maps and generates global attention weights via a Split-Excite Layer [25,26]. Concurrently, it performs a partial channel exchange between the inputs and integrates the exchanged features with the difference map to derive spatial attention weights. These weights are then applied to the original input feature maps for refinement. Finally, the two enhanced feature maps are concatenated and processed through a depthwise separable convolution to yield the final fusion result.

Given the bi-temporal features of the i-th layer, denoted as $F_{1}^{i}$ and $F_{2}^{i}$, an element-wise subtraction is first performed to generate a coarse change representation, $F_{d}^{i}$. Subsequently, these features undergo channel-wise concatenation via the Channel Exchange (CE) module [13] to yield $F_{12}^{i}$ and $F_{21}^{i}$, respectively. This operation facilitates cross-channel interaction between the two temporal phases prior to explicit subtraction, thereby accentuating variations while attenuating invariant regions. Next, the features obtained by integrating $F_{d}^{i}$ with $F_{12}^{i}$ and $F_{21}^{i}$ are independently processed through depthwise separable convolutions (*DSConv*) and wavelet convolutions (*WtConv*) [27]. The output of the *WtConv* layer is subjected to a sigmoid activation function to generate weight matrices $W_{1}^{i}$ and $W_{2}^{i}$, which are then combined. This design aims to reduce the parameter count while improving the feature extraction efficacy of the network. The process can be formulated as follows:

(1) $$W_{j}^{i}=WtConv\left[DSConv\left(F_{d}⊕F_{jk}\right)\right],j\in \left\{1,2\right\},k\in \left\{1,2\right\}$$

where ⊕ denotes concatenation. Subsequently, *σ* denotes the sigmoid activation function.

To further enhance the expressive power of multi-scale features, we introduce a SELayer to process the initial difference $F_{d}^{i}$, aiming to capture richer inter-channel dependencies. Its formula is expressed as

(2) $$W_{S}^{i}=\sigma (\sum_{k}\;(\Psi_{k}(pooling(F_{d}))))$$

where $\Psi_{k}$ denotes a Dense Multi-Layer Perceptron (DMLP), which consists of a linear mapping and a ReLU activation function, while $\sum_{k}$ represents connections along the channel, in our model, *k* is set to 4. This module leverages global knowledge to enhance more discriminative features in regions that vary over the temporal dimension.

Finally, we apply the spatially augmented weight matrices $W_{j}^{i}$ and the attention weights $W_{S}^{i}$ derived from the SELayer to the source feature map $F_{j}^{i}$, yielding the fusion result $F_{diff}^{i}$:

(3) $$F_{diff}^{i}=\left(W_{S}^{i}⊗W_{1}^{i}⊗F_{1}^{i}\right)⊕\left(W_{S}^{i}⊗W_{2}^{i}⊗F_{2}^{i}\right)$$

where ⊗ denotes element-wise multiplication. Finally, the feature maps obtained from the concatenation branch and the subtraction branch are fused:

(4) $$X_{i}=F_{cat}^{i}+F_{diff}^{i}$$

Obtain the final fusion result $X_{i}$ from DSCF for subsequent module processing.

### 2.3. Deep-Guided Multiscale Decoder (DGMD)

In change detection tasks, shallow features retain rich spatial details and boundary information, while deep features exhibit stronger semantic representation capabilities, facilitating the characterization of the overall distribution and change trends of mangrove forests. However, in mangrove scenarios subject to significant tidal interference, features at different scales demonstrate inherent inconsistencies in their response to change: shallow features are vulnerable to noise induced by water inundation and boundary displacement, whereas deep features may fail to capture local change details due to their reduced resolution. Consequently, direct cross-scale fusion may introduce redundant information and potentially compromise the model’s efficacy in identifying genuine change areas.

To address these challenges, we propose the Deep-Guided Multiscale Decoder (DGMD), illustrated in Figure 2, which comprises a Deep Feature Guidance (DFG) module, a Deep Feature Guided Fusion (DFGF) module, and a Feature Refinement (FR) module. The DFG module leverages high-level semantic information from two distinct scales integrated with a self-attention mechanism to generate a guidance map, denoted as $F_{DFG}$, characterizing stable and discriminative change regions. Subsequently, the DFGF module utilizes $F_{DFG}$ to facilitate cross-scale feature fusion, aligning shallow and deep features within change regions to promote complementary learning. This strategy mitigates tidal-induced boundary perturbations while preserving critical spatial details. Following processing by the FR module, the fused features from each layer demonstrate enhanced saliency in high-change areas, retaining rich spatial information while suppressing irrelevant variations. Consequently, this framework enables a refined characterization of mangrove distribution changes.

In contrast to MSFCTNet [28], which employs a representative multi-scale decoder architecture by concatenating features from four encoding stages, processing them via a Hybrid Convolutional Neural Network–Transformer Module (HCTM), and filtering cross-scale information through a Gated Attention Module (GAM), our approach presents several distinct architectural advantages.

First, while MSFCTNet executes full-scale feature aggregation followed by global processing, our DGMD implements a progressive decoding strategy that iteratively refines features in a layer-by-layer fashion. Second, the GAM in MSFCTNet leverages SE-based attention to perform implicit channel reweighting across scales; conversely, our DFG module constructs explicit semantic guidance maps derived from the two deepest features ($X_{3}$ and $X_{4}$) to direct feature enhancement, thereby avoiding the uniform treatment of all scales. This design promotes more targeted refinement of change regions and aids in the preservation of mangrove boundaries amidst tidal disturbances. The quantitative comparison between MSFCTNet and our DGMD is presented in Table 3.

#### 2.3.1. Deep Feature Guided (DFG)

**Figure 5:**
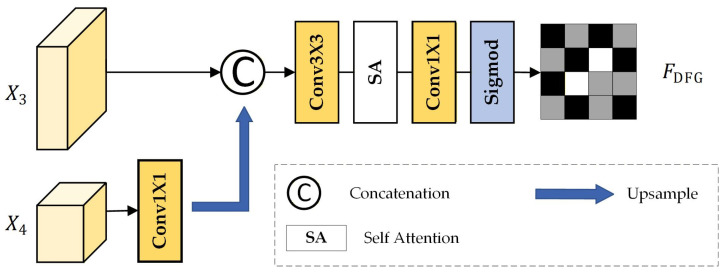
The architecture of the DFG module.

The architecture of the proposed DFG module is shown in Figure 5. The input features are $X_{3}\;\in R^{256\times H/16\times W/16}$ and $X_{4}\;\in R^{512\times H/32\times W/32}$. The output is $F_{DFG}\;\in R^{1\times H/16\times W/16}$.

Considering the differing spatial and channel dimensions of $X_{3}$ and $X_{4}$, upsampling and 1 × 1 convolution are employed to align their spatial scales. The channel dimension of the high-level features is compressed, after which the two feature maps at the same spatial scale are concatenated. These concatenated features are then further processed using 3 × 3 convolution operations to enhance feature representation. The formalized process of this operation is as follows:

(5) $$F_{Conv}={Conv}_{3\times 3}(X_{3}⊕Up({Conv}_{1\times 1}\left(X_{4}\right)))$$

where up denotes the upsampling operation, ${Conv}_{i\times i}$ represents a *i* × *i* convolution.

Due to the limited receptive field of the 3 × 3 convolution kernel, the output correlates only with a local portion of the input, resulting in sufficient global contextual information. To address this, a self-attention mechanism is employed in the subsequent processing. Next, a 1 × 1 convolution reduces the number of channels to one, aiming to fuse multi-dimensional feature information into a compact representation for further processing. Finally, the sigmoid activation function generates the DFG map:

(6) $$F_{DFG}=\sigma \left({Conv}_{1\times 1}(SA(F_{Conv}))\right)$$

where SA denotes the self-attention mechanism, and $F_{DFG}$ represents the output DFG mapping.

This process ensures the DFG mapping effectively integrates local and global feature information, enhancing the representational capacity of the DFG mapping.

#### 2.3.2. Deep Feature Guided Fusion (DFGF)

**Figure 6:**
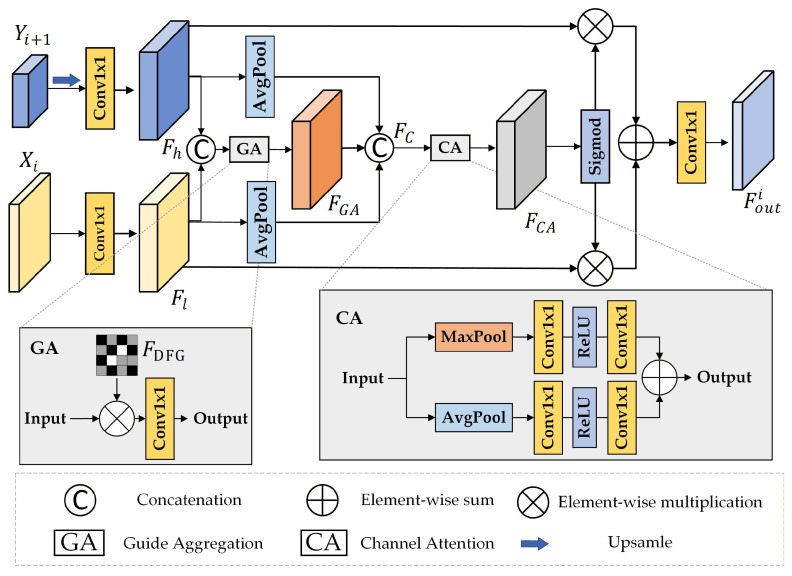
The architecture of the DFGF module.

The architecture of the proposed DFGF module is shown in Figure 6. This module is designed to perform multiscale feature fusion using the generated $F_{DFG}$. It first upsamples the fusion result from the previous layer and adjusts its channels to align with the shallow features in spatial resolution. Next, it expands the channels of both features and performs gated weighted fusion guided by $F_{DFG}$. Global context information is then introduced and concatenated with the fused features, followed by channel attention and spatial selection mechanisms to generate adaptive weights. Finally, these weights are used to perform weighted fusion of the original features, and the result is passed through a projection layer to produce the final output.

(7) $$F_{h}={Conv}_{1\times 1}[Up(Y_{i+1})]$$

(8) $$F_{l}={Conv}_{1\times 1}(X_{i})$$

Subsequently, the processed features are concatenated along the channel dimension. Concurrently, the guidance map $F_{DFG}$ is processed by a Guide Aggregation (GA) layer, in which each element is subjected to a sigmoid activation followed by the addition of a unit value (i.e., +1). This strategy enhances the preservation of original features, mitigating information loss attributed to potential instability within the guidance map. The GA layer then applies element-wise multiplication to the two processed features, as formulated below. The resulting feature map $F_{GA}$ possesses dimensions of ${2C}^{\prime }\times H^{\prime }\times W^{\prime }$:

(9) $$F_{GA}={Conv}_{1\times 1}[(F_{h}⊕F_{L})⊗(\sigma F_{DFG}+1)]$$

where the ⊗ denotes element-wise multiplication.

In parallel with this step, the processed deep features are combined with shallow features via global features, which are then concatenated with $F_{GA}$:

(10) $$F_{C}=Avg(F_{h})⊕Avg(F_{l})⊕F_{GA}$$

where *Avg* denotes the operation of computing the average value of the feature map and extending it across the entire pixel space. This operation aims to obtain a global feature capturing the overall properties of the input features.

The concatenated feature map $F_{C}\in R^{{6C}^{\prime }\times H^{\prime }\times W^{\prime }}$ is subsequently input into the Channel Attention (CA) layer, which executes global channel-level filtering and recalibration on the fused features, yielding $F_{CA}\in R^{{6C}^{\prime }\times H^{\prime }\times W^{\prime }}$. This mechanism ensures that the features propagated to the decoder exhibit enhanced compactness and discriminability, thereby improving change detection accuracy. The process is formulated as follows:

(11) $$F_{CA}=FC[Max(F_{C})]+FC[Avg(F_{C})]$$

where *FC* denotes the fully connected layer, and *Max* represents max pooling.

Finally, we apply a Sigmoid activation to $F_{CA}$, then multiply it with the shallow features $F_{l}$ and the deep fusion features $F_{h}$ respectively, and sum the results to obtain the fusion output:

(12) $$F_{out}^{i}=(\sigma F_{CA}⊗F_{l)}+(\sigma F_{CA}⊗F_{h})$$

The dimension of $F_{out}^{i}$ is $C^{\prime }\times H^{\prime }\times W^{\prime }$.

The Deep Feature Guided Fusion (DFGF) module takes as input the current layer’s features $X_{i}\in R^{C^{\prime }\times H^{\prime }\times W^{\prime }}$, the fused features $Y_{i+1}\in R^{2C^{\prime }\times \frac{H^{\prime }}{2}\times \frac{W^{\prime }}{2}}$ derived from the preceding layer, and the guidance map $F_{DFG}\in R^{1\times \frac{H}{16}\times \frac{W}{16}}$. Initially, $Y_{i+1}$ is upsampled to match the spatial resolution of $X_{i}$. Subsequently, a 1×1 convolution is applied to expand the channel dimension of both feature maps by a factor of two, yielding $F_{h},F_{l}\in R^{{2C}^{\prime }\times H^{\prime }\times W^{\prime }}$.

### 2.4. Loss Function

In remote sensing change detection, changed pixels typically comprise only a negligible fraction of the image, leading to a severe class imbalance between changed and unchanged regions. This disparity causes standard cross-entropy loss to be dominated by the prevalent unchanged pixels, thereby obscuring the learning signals critical for change regions. To mitigate this issue, we propose a hybrid loss function that integrates Focal Loss and Dice Loss. Focal Loss dynamically reweights samples to prioritize hard-to-classify pixels, whereas Dice Loss maximizes the spatial overlap between predicted segments and ground truth labels. This synergistic combination effectively alleviates the class imbalance problem, steering the model toward more precise identification of change areas. The formulation is presented as follows:

(13) $$L=L_{focal}+L_{dice}$$

Among these, $L_{focal}$ denotes the focal loss, formulated as

(14) $$L_{focal}=-\alpha (1-\hat{p}{)}^{\gamma }log(\hat{p})$$

(15) $$\hat{p}=\left\{\begin{array}{ll} p, & \mathrm{if}\;y=1\\ 1-p, & \mathrm{otherwise} \end{array}\right.$$

Among these, hyperparameters α (set to 0.2) and *γ* (set to 2) are used to balance the weights of positive and negative samples and focus on difficult-to-detect samples, respectively; *p* represents the prediction probability, and *y* denotes the binary label (0 or 1) indicating whether a pixel has changed or remained unchanged.

$L_{dice}$ represents the loss from dice rolls, calculated using the following formula:

(16) $$L_{dice}=1-\frac{2\cdot E\cdot softmax\left(E^{\prime }\right)}{E+softmax\left(E^{\prime }\right)}$$

(17) $$E^{\prime }=\left\{e_{k}^{\prime },k=1,2,\ldots ,H\times W\right\}$$

where *E* denotes the ground truth, $E^{\prime }$ represents the change map, and $e_{k}^{\prime }$ indicates a point within $E^{\prime }$.

## 3. Experiments and Analysis

### 3.1. Datasets

We systematically evaluated the performance of the proposed DSDGMNet for mangrove change detection. The core experiment was conducted on the mangrove change detection dataset GBCNR, independently compiled in our laboratory. Additionally, we performed supplementary validation on several public datasets: LEVIR-CD [29], WHU [30], CLCD [31], and SYSU-CD [32].

**Figure 7:**
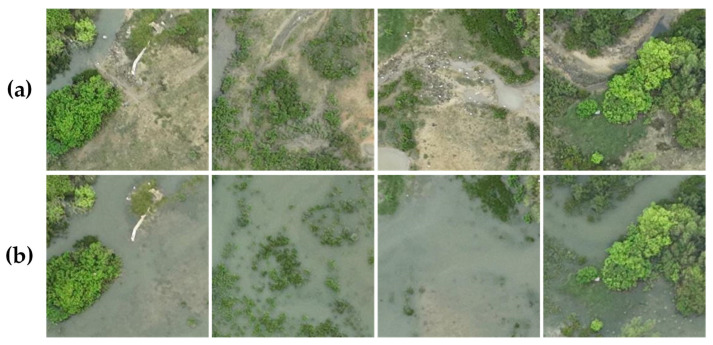
GBCNR Dataset Examples: (**a**) at Time T1, (**b**) at Time T2.

GBCNR: The Guangxi Coastal National Wetland Reserve Mangrove Tidal Change Detection Dataset (GBCNR) was collected using DJI Mavic series UAVs (Shenzhen DJI Innovation Technology Co., Ltd., Shenzhen, China) along the Fengjia River in Beihai, Guangxi, China. Representative mangrove areas were selected, and multiple surveys were conducted under different tidal conditions. The UAVs operated at altitudes up to 500 m, and each image has a resolution of 5280 × 3956 pixels. Under the guidance of environmental ecology experts, wetland areas were annotated using LabelMe. The images were cropped into tiles and resized to 256 × 256 pixels to meet deep learning input requirements. Data augmentation, including horizontal and vertical flipping, was applied. The dataset was divided into 1748 training pairs, 499 validation pairs, and 249 test pairs. Examples of the GBCNR dataset under different tidal conditions are shown in Figure 7, where (a) and (b) represent the same location at Time T1 and Time T2, respectively.

LEVIR-CD: A building change detection dataset containing 637 image pairs (0.5 m resolution, 1024 × 1024 pixels) from 20 regions in Texas, USA. We cropped them into 256 × 256 non-overlapping patches, resulting in 7120/1024/2048 patches for training/validation/test.

WHU: Consists of a single pair of aerial images (0.2 m resolution, 32,507 × 15,354 pixels) capturing earthquake-induced building changes. We cropped it into 256 × 256 non-overlapping patches, yielding 6095/737/787 patches for training/validation/test.

CLCD: A land cover change detection dataset based on Landsat imagery (30 m resolution) covering China from 1985 onward. Images are provided as 512 × 512 tiles (each covering ~15.36 km^2^). We used the official split of 360/120/120 for training/validation/test.

SYSU-CD: Contains high-resolution optical images (0.5 m resolution, 256 × 256 patches) from Shenzhen, China, covering various urban change types. The dataset is split into 12,000/4000/4000 patches for training/validation/test.

**Figure 8:**
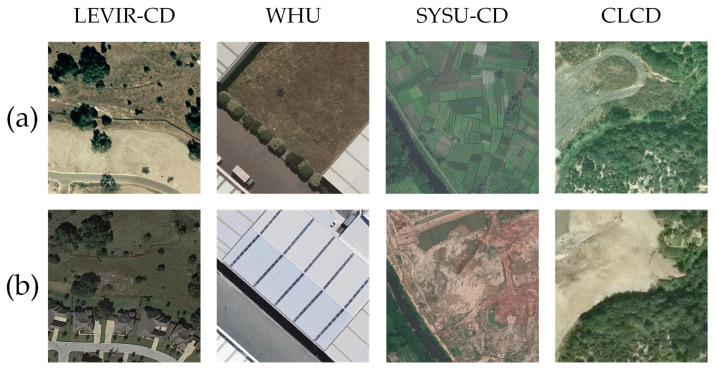
Some Dataset Examples: (**a**) at Time T1, (**b**) at Time T2.

Figure 8 shows representative image pairs from these datasets, with (a) and (b) corresponding to Time T1 and Time T2 for each example.

### 3.2. Quality Control

The GBCNR dataset was constructed for mangrove change detection using high-resolution satellite and UAV imagery. This section details the annotation protocol and quality control measures.

#### 3.2.1. Annotation Protocol

All annotations were conducted by three remote sensing experts, each possessing more than five years of experience in wetland interpretation. Utilizing ArcGIS (version 10.8.2) and LabelMe (version 5.8.2) software, the annotators adhered to a rigorous temporal contrast annotation protocol comprising the following steps: (1) Image Registration: Bi-temporal UAV images were precisely aligned using the Scale-Invariant Feature Transform (SIFT) algorithm [33] to rectify positional discrepancies; (2) Change Delineation: Experts manually mapped mangrove changes—including expansion, degradation, and anthropogenic disturbances—by conducting a comparative analysis of the registered image pairs, augmented by NDVI and NDWI indices for spectral verification.

#### 3.2.2. Quality Control and Inter-Annotator Consistency

To guarantee annotation reliability, we implemented a rigorous two-stage cross-validation strategy:

(1) Independent Annotation: Each image pair was independently labeled by two experts. Both annotators adhered to the identical protocol and were blinded to each other’s results during this phase.
(2) Disagreement Adjudication: All annotations from Stage 1 were subjected to pixel-wise comparison. For discrepant pixels, a third senior expert conducted a review and rendered the final verdict. This adjudication process ensured the consistent resolution of all ambiguous cases.

Inter-annotator agreement was quantitatively evaluated using Cohen’s Kappa coefficient. The average Kappa score across all samples achieved 0.87, indicating substantial agreement. Consequently, only samples exhibiting a Kappa score exceeding 0.85 were included in the final dataset. The quality control metrics and standards used in this annotation process are summarized in Table 4. SSIM means Structural Similarity Index Measure.

#### 3.2.3. Dataset Variability

The dataset comprises imagery acquired between 2023 and 2024, facilitating the observation of subtle dynamic changes within mangrove ecosystems. To explicitly address tidal variability, flight missions were strategically scheduled in accordance with tidal predictions at two distinct tidal regimes: 5.11 m (high tide) and 0.23 m (low tide). This approach ensures comprehensive representation of ecological characteristics across diverse tidal conditions.

### 3.3. Implementation Details and Metrics

#### 3.3.1. Implementation Details

We implemented our method using the PyTorch (version 2.0.0) framework on an NVIDIA GeForce RTX 3090 GPU with 24 GB of memory. The model was trained for 200 epochs using the SGD optimizer with an initial learning rate of 0.05, momentum of 0.9, and weight decay of 5 × 10^−5^. A step learning rate decay strategy was adopted, reducing the learning rate by a factor of 0.1 every 50 epochs.

For the loss function, we employed a combination of Focal Loss and Dice Loss with equal weights (0.5 each). The Focal Loss was configured with γ = 0 and without class weighting (α = None), while Dice Loss included an epsilon value of 1 × 10^−7^ for numerical stability.

Input images were resized to 256 × 256 pixels for the GBCNR, LEVIR-CD, WHU-CD, and SYSU-CD datasets, and to 512 × 512 pixels for CLCD. The batch size was set to 8 for all datasets except CLCD, where a batch size of 4 was used due to the larger input resolution and GPU memory constraints.

Data augmentation was applied to the bi-temporal images. On the GBCNR dataset, random rotation (probability = 0.15), vertical flip (probability = 0.3), and horizontal flip (probability = 0.5) were performed.

To ensure reproducibility, all experiments except the statistical evaluation in Section 3.6 were conducted with a fixed random seed of 1234. For statistical evaluation, five different random seeds (1234, 2345, 3456, 4567, and 5678) were used to report the mean and standard deviation.

#### 3.3.2. Metrics

To quantitatively assess the effectiveness of the CD method, we employed five widely used metrics to compare the predicted change map with the actual situation. These include Precision (Pre), Recall (Rec), F1 Score (*F*1), and Intersection over Union (IoU). Their definitions are as follows:

(18) $$Pre=\frac{TP}{TP+FP},Rec=\frac{TP}{TP+FN}$$

(19) $$F1=\frac{2\times Pre\times Rec}{Pre+Rec},\;IoU=\frac{TP}{TP+FP+FN}$$

TP, TN, FP, and FN represent the number of true positives, true negatives, false positives, and false negatives, respectively. Their values range from 0 to 1, with higher values indicating better performance.

#### 3.3.3. Computational Efficiency

We evaluated the training time and inference speed of our method. All models were trained for 200 epochs. Table 5 summarizes the training time across datasets.

For inference, we measured the processing speed on the WHU-CD test set (787 images, 256 × 256). Our model processes a single image in 11.8 ms, equivalent to 84.8 FPS. Since all 256 × 256 datasets share the same input resolution, the inference speed is consistent across them. For the CLCD dataset with 512 × 512 resolution, inference time scales approximately at 45.7 ms per image, equivalent to 21.9 FPS.

### 3.4. Ablation Experiments

#### 3.4.1. Effect of Core Modules

To validate the effectiveness of the core modules proposed in this paper, we conducted systematic ablation experiments across five datasets. The experiments included a baseline model STNet [24] with no modified modules, a model incorporating the DSCF module, a variant without the DFG module (denoted as DSDGMNet w/o DFG) and the full DSDGMNet. Results are shown in Table 6.

Ablation experiments across five datasets demonstrate that both DSCF and DGMD contribute positively to model performance. The baseline model achieves F1 scores ranging from 51.54% to 90.42% across datasets. Adding DSCF alone yields consistent improvements (0.09–1.53%), while DGMD alone produces larger gains, particularly on GBCNR (+19.82%), highlighting its critical role in multiscale feature fusion for mangrove change detection. Removing the DFG module leads to performance degradation across all datasets (e.g., 1.01% on GBCNR and 0.61% on WHU), confirming its effectiveness in feature guidance. The full model combining both modules achieves the highest F1 scores on all datasets, with the most notable improvements on WHU-CD (+1.66%) and CLCD (+1.77%). These results validate that DSCF and DGMD complement each other, and their integration is essential for optimal performance across diverse change detection scenarios.

#### 3.4.2. Effect of Loss Functions

We conducted a comparative study to evaluate the effect of different loss function configurations on model performance. Specifically, three variants were examined: (1) using Focal loss alone, (2) using Dice loss alone, and (3) employing a combined loss function (Focal + Dice), as implemented in our full model. All experiments were carried out on the GBCNR dataset, and the results are summarized in Table 7.

The combined loss achieves the best performance (F1: 71.36%, IoU: 55.47%), outperforming focal-only (F1: 69.35%, IoU: 53.08%) and Dice-only (F1: 69.89%, IoU: 53.71%). The combined loss yields significantly higher recall (84.57% vs. 76.06%/77.35%), indicating its effectiveness in identifying change regions. These results confirm that both loss components contribute positively to mangrove change detection.

#### 3.4.3. Effect of Backbone Networks

We assessed the performance of three backbone architectures under consistent experimental settings on the GBCNR dataset: ResNet-18 (adopted as our default), ResNet-34, and ResNet-50. All experiments were executed in a single run. Furthermore, we quantified computational complexity via floating-point operations (FLOPs, in G) and parameter count (Params, in M) to evaluate efficiency.

The results, presented in Table 8, reveal that ResNet-18 yields the optimal overall performance, achieving an F1-score of 71.36% and an IoU of 55.47%, thereby surpassing both deeper variants by a significant margin. Specifically, relative to ResNet-34 (F1: 68.83%, IoU: 52.48%), ResNet-18 delivers gains of 2.53% in F1 and 2.99% in IoU; similarly, against ResNet-50 (F1: 68.69%, IoU: 52.31%), it registers improvements of 2.67% in F1 and 3.16% in IoU. Notably, ResNet-18 incurs only 12.84 G FLOPs and 38.09 M parameters—figures substantially lower than those of ResNet-34 (17.68 G, 48.21 M) and ResNet-50 (19.40 G, 51.82 M)—underscoring its superior trade-off between accuracy and computational efficiency.

These results indicate that deeper backbone architectures do not necessarily enhance mangrove change detection performance on our dataset. This phenomenon can be attributed to the fact that increased model capacity may induce overfitting given the relatively limited scale of the GBCNR dataset. Moreover, mangrove change detection hinges critically on precise boundary localization rather than solely on deep semantic abstraction. Shallower architectures, such as ResNet-18, are more effective at preserving fine spatial details in early feature layers, which are paramount for accurately delineating tidally influenced boundaries. Consequently, ResNet-18 is selected as the default backbone, providing an optimal balance between accuracy and computational efficiency.

### 3.5. Comparative Experiments

To comprehensively evaluate the performance and generalization capabilities of our proposed DSDGMNet model, we conducted extensive experiments on four publicly available remote sensing change detection datasets and the GBCNR dataset. We selected multiple state-of-the-art methods for comparison, including: FC-EF (Fully Convolutional Early Fusion), FC-Siam-diff (Fully Convolutional Siamese Networks with Difference), FC-Siam-conc (Fully Convolutional Siamese Networks with Concatenation) [10], LGPNet [34], SNUNet [35], ChangeFormer [19], USSFCNet [36], and DDL-Net [37]. All experiments were performed under identical data partitioning and hardware/software environments to ensure fairness.

As shown in Table 9, our method achieves the highest recall (84.57%), F1 (71.36%), and IoU (55.47%) on the GBCNR dataset. Compared to multi-scale methods like SNUNet (F1: 68.87%) and LGPNet (F1: 69.13%), and Transformer-based approaches like ChangeFormer (F1: 66.39%), our model improves F1 by 2.2–4.9%. The precision (61.71%) remains comparable to other methods, indicating that the gain in recall does not come at the cost of excessive false alarms. This demonstrates the effectiveness of our tide-aware design in complex mangrove environments.

On the LEVIR-CD dataset, our method achieved the best overall performance (Table 10), with an F1 score of 90.68% and an IoU of 82.95%. The model achieves a balance trade-off between Pr (91.36%) and Re (90.01%), avoiding the imbalance issues seen in methods like FC-Siam-diff (high precision, low recall) or FC-Siam-conc (high recall, low precision). This is primarily attributed to the core dual-stream change fusion module, which enhances regions of genuine change through difference-aware attention while supplementing details via the connection branch. Consequently, it outperforms competitors like ChangeFormer and DDLNet in building change detection.

On the WHU dataset, our model demonstrates even more pronounced advantages (Table 11), achieving an F1 score of 91.38%—outperforming other methods and surpassing the second-place DDLNet by 1.53 percentage points. The model simultaneously achieves the highest Re (91.85%) and a very high Pr (90.92%), indicating its ability to detect nearly all changed buildings while effectively controlling false positives. Its IoU (84.13%) also ranks first, suggesting that the deep feature-guided fusion module effectively utilizes deep semantic guidance for multiscale Feature Fusion, enhancing change regions while suppressing background redundancy.

The SYSU-CD dataset encompasses diverse changes across buildings, roads, water bodies, and other elements, presenting complex scenes. As shown in Table 12, all models exhibit performance degradation, reflecting the dataset’s challenging nature. Despite this, our approach ranked first with an F1 score of 82.43% and an IoU of 70.11%, outperforming the second-place DDLNet by 1.01% and 1.45%, respectively. The model also achieved the highest Pr (85.89%), highlighting its ability to suppress false changes in complex terrain. This is attributed to the multi-scale guided fusion architecture, which integrates deep semantic information with shallow details within a global context, thereby improving decision reliability.

The CLCD dataset features diverse targets, complex backgrounds, and significant noise, and the resolution (512 × 512) set for this experiment further increases detection difficulty. As shown in Table 13, all models perform noticeably worse than on other datasets. In this highly challenging environment, our model once again demonstrates robustness, achieving an F1 score of 75.31%—0.82% higher than the second-place DDLNet and 1.04% higher in IoU. The model achieved the highest Re (74.65%) while maintaining high Pr (75.98%), indicating its ability to capture subtle, irregular changes in targets such as farmland. The dual-stream change fusion module sensitively responds to weak difference signals and effectively locates genuine changes within complex backgrounds.

### 3.6. Statistical Evaluation

To evaluate the stability and robustness of the proposed method, we conducted five independent runs on our self-constructed GBCNR dataset and the publicly available WHU dataset using different random seeds (1234, 2345, 3456, 4567, 5678) [31]. All results of our method are reported as mean ± standard deviation, providing a more reliable assessment of the model’s performance under different initialization conditions. The results are shown in Table 14. Due to computational constraints, all competing methods were evaluated with a single run.

### 3.7. Visualization of Comparison Experiments

#### 3.7.1. Qualitative Comparison on Representative Scenes

To more intuitively demonstrate the effectiveness of the proposed method, this section will analyze and discuss the visualization results and model efficiency of the comparative experiments.

**Figure 9:**
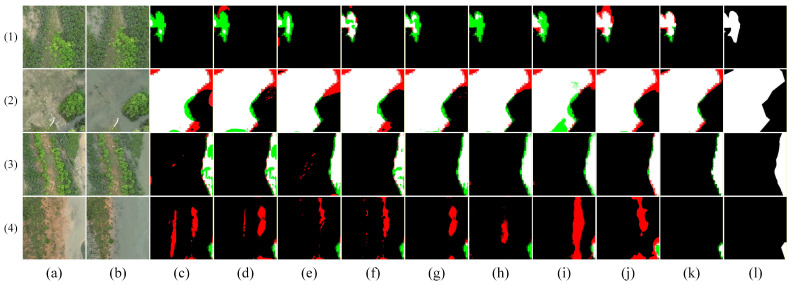
Visual comparison of change detection results on the GBCNR dataset. (**a**) T1 image; (**b**) T2 image; (**c**) FC-EF; (**d**) FC-Siam-diff; (**e**) FC-Siam-conc; (**f**) LGPNet; (**g**) SNUNet; (**h**) ChangeFormer; (**i**) USSFC-Net; (**j**) DDL-Net; (**k**) Ours; (**l**) Ground truth. In the change maps, white represents true positives, black represents true negatives, red represents false positives, and green represents false negatives.

**Figure 10:**
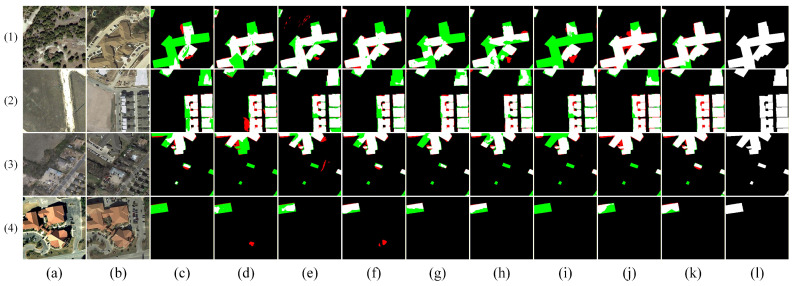
Visual comparison of change detection results on the LEVIR-CD dataset. (**a**) T1 image; (**b**) T2 image; (**c**) FC-EF; (**d**) FC-Siam-diff; (**e**) FC-Siam-conc; (**f**) LGPNet; (**g**) SNUNet; (**h**) ChangeFormer; (**i**) USSFC-Net; (**j**) DDL-Net; (**k**) Ours; (**l**) Ground truth. In the change maps, white represents true positives, black represents true negatives, red represents false positives, and green represents false negatives.

**Figure 11:**
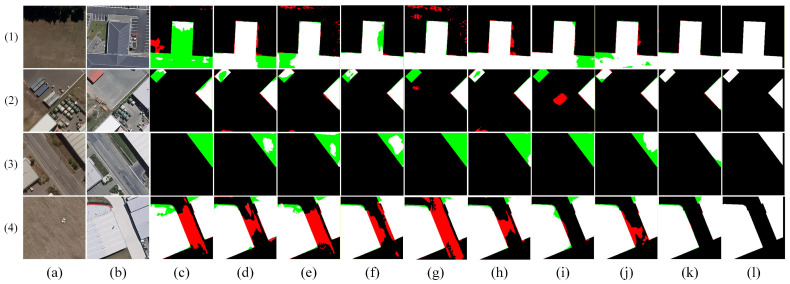
Visual comparison of change detection results on the WHU-CD dataset. (**a**) T1 image; (**b**) T2 image; (**c**) FC-EF; (**d**) FC-Siam-diff; (**e**) FC-Siam-conc; (**f**) LGPNet; (**g**) SNUNet; (**h**) ChangeFormer; (**i**) USSFC-Net; (**j**) DDL-Net; (**k**) Ours; (**l**) Ground truth. In the change maps, white represents true positives, black represents true negatives, red represents false positives, and green represents false negatives.

**Figure 12:**
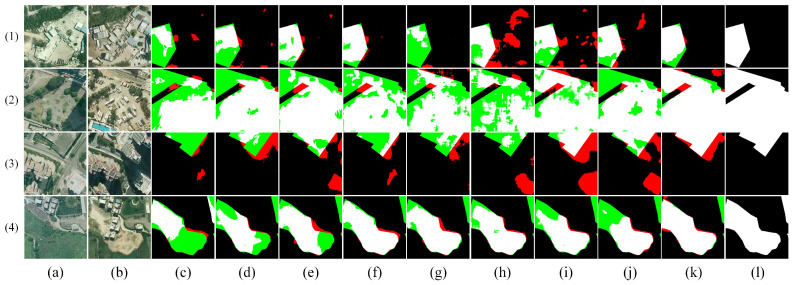
Visual comparison of change detection results on the SYSU-CD dataset. (**a**) T1 image; (**b**) T2 image; (**c**) FC-EF; (**d**) FC-Siam-diff; (**e**) FC-Siam-conc; (**f**) LGPNet; (**g**) SNUNet; (**h**) ChangeFormer; (**i**) USSFC-Net; (**j**) DDL-Net; (**k**) Ours; (**l**) Ground truth. In the change maps, white represents true positives, black represents true negatives, red represents false positives, and green represents false negatives.

**Figure 13:**
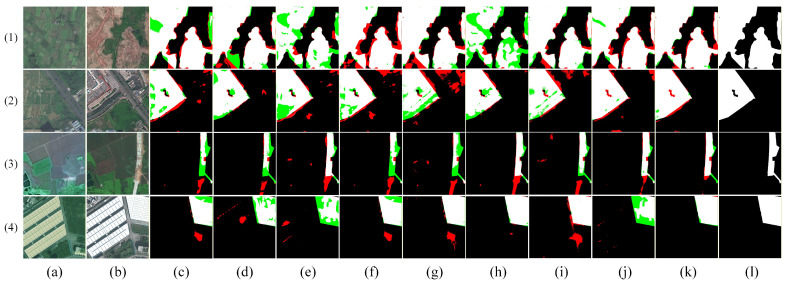
Visual comparison of change detection results on the CLCD dataset. (**a**) T1 image; (**b**) T2 image; (**c**) FC-EF; (**d**) FC-Siam-diff; (**e**) FC-Siam-conc; (**f**) LGPNet; (**g**) SNUNet; (**h**) ChangeFormer; (**i**) USSFC-Net; (**j**) DDL-Net; (**k**) Ours; (**l**) Ground truth. In the change maps, white represents true positives, black represents true negatives, red represents false positives, and green represents false negatives.

Figure 9, Figure 10, Figure 11, Figure 12 and Figure 13 show the visual results of randomly selecting four pairs of remote sensing images from each of the five databases.

In the figure, white represents true positives, black represents true negatives, red represents false positives, and green represents false negatives. The labels in the figure denote: (a) T_1_ image, (b) T_2_ image, (c) FC-EF [10], (d) FC-Siam-diff [10], (e) FC-Siam-conc [10], (f) LGPNet [34], (g) SNUNet [35], (h) ChangeFormer [19], (i) USSFC-Net [36], (j) DDL-Net [37], (k) Ours, and (l) Ground Truth.

As shown in Figure 9, visualization results on the GBCNR dataset provide intuitive validation of the proposed network’s effectiveness in addressing the two core challenges of mangrove change detection. Regarding the issue of apparent changes under tidal disturbances while mangrove structures remain stable, examples (1) and (3) reveal that most comparison methods (e.g., d–k) tend to misinterpret changes in water body extent and tidal channel morphology as mangrove changes, generating extensive false positives. In contrast, our method (l) accurately focuses on genuine mangrove distribution changes while significantly suppressing false responses caused by tides, water surface reflections, and other factors. Addressing the challenge of inconsistent change scales and highly unstable boundaries, in the more complex examples (2) and (4), the proposed method maintains overall semantic consistency of change areas while clearly delineating fine-grained boundary structures at mangrove–water interfaces. This effectively reduces fragmented noise and boundary blurring. Collectively, these results demonstrate that our method achieves robust and adaptable performance in coastal wetland scenarios with significant tidal interference, simultaneously ensuring accurate modeling of real-world changes and stable representation of multi-scale boundary details.

As shown in Figure 10, the visualization comparison on the LEVIR-CD test set demonstrates that DSDGMNet exhibits outstanding performance across multiple typical scenarios. For instance, in scenario (1), compared to the fragmented results produced by FC-EF, FC-Siam-diff, and FC-Siam-conc, DSDGMNet achieves complete coverage of the building area while maintaining shape consistency. For complex-contoured buildings (3), its boundaries are the clearest, outperforming SNUNet and ChangeFormer; in road and shadow interference scenarios (2), DSDGMNet effectively suppresses false detections seen in BIT and DDLNet; and for sparse, small-scale building detection (4), it successfully captures changes missed by other methods. Overall, DSDGMNet achieves higher integrity, accuracy, and robustness in building change detection.

The visualization results on the WHU-CD dataset (Figure 11) clearly demonstrate that our proposed DSDGMNet achieves outstanding performance in building change detection. As shown in examples (1) and (3), our method (k) can completely reconstruct the building contours, significantly reducing the internal voids (false negatives) and boundary defects observed in other methods (e.g., c–j). Simultaneously, in the complex backgrounds of examples (2) and (4), our model nearly completely suppresses false detections in non-building areas (e.g., open spaces, vegetation), generating the cleanest and most accurate change maps. This fully demonstrates DSDGMNet’s dual advantages in achieving high-integrity detection and high-precision localization.

In the visual comparison of the SYSU-CD dataset (Figure 12), DSDGMNet demonstrates outstanding performance in complex multi-class change detection tasks. For instance, in mixed change scenarios involving roads, vegetation, and buildings (Examples 1 and 2), methods like BIT and SNUNet exhibit significant false detections, whereas DSDGMNet accurately distinguishes genuine changes from background interference, generating the cleanest change maps. Regarding change region integrity (Examples 3 and 4), DSDGMNet effectively preserves change region continuity through Multiscale Feature Fusion, overcoming the false negative issues observed in FC-Siam-diff and USSFC-Net. Simultaneously, it achieves the highest precision in boundary localization, producing clear and natural contours. Overall, DSDGMNet demonstrates superior detection reliability and robustness in multi-category complex scenarios.

The visualization results on the CLCD dataset (Figure 13) fully demonstrate the exceptional robustness of our proposed DSDGMNet in challenging scenarios. As shown in examples (1) and (2), our method effectively suppresses false detections when confronted with complex farmland variations and substantial noise interference, significantly outperforming other comparative approaches. Similarly, in examples (3) and (4), our model achieves more complete identification of genuine change areas, effectively reducing missed detections. These results demonstrate its robust capability to accurately distinguish real changes from background noise in challenging scenarios.

#### 3.7.2. Challenging and Failure Case Analysis

**Figure 14:**
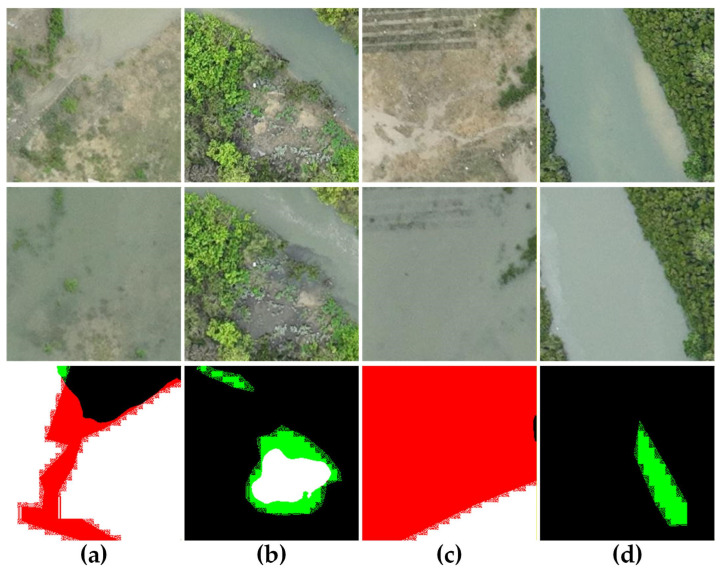
Visualization of challenging and failure cases on the GBCNR dataset. (**a**–**d**) Four representative failure scenarios under extreme tidal variations and boundary drift. For each case, the subfigures (**top** to **bottom**) show: T1, T2, Ours. Color scheme: white (TP), black (TN), red (FP), green (FN).

To further assess the robustness of the proposed method, several challenging and failure cases on the GBCNR dataset are presented in Figure 14. These examples illustrate the limitations of the compared methods under extreme tidal variations and boundary drift conditions.

In Cases (a) and (c), significant tidal inundation causes large spectral variations while the mangrove structure remains unchanged, leading to false positives in appearance-driven models. In Cases (b) and (d), boundary drift and narrow mangrove strips result in missed detections, highlighting the difficulty of capturing fine-scale changes under strong class imbalance. Although the proposed method suppresses a large portion of tidal-induced pseudo-changes, residual errors persist under extremely complex hydrological conditions, suggesting directions for future research.

### 3.8. Model Efficiency

To comprehensively evaluate the practicality of the proposed method, we conducted a comparative analysis of the number of parameters (Params) and computational complexity (FLOPs) of various models on the GBCNR dataset, with input image size standardized to 256 × 256 × 3 and batch size set to 1, as shown in Table 15. DSDGMNet has 38.09 M parameters and 12.84 G floating-point operations. Compared with current mainstream models, our method achieves a good balance between computational efficiency and detection accuracy. Specifically, compared with the similarly performing ChangeFormer (202.78 G FLOPs, 41.03 M Params), our method reduces computation by approximately 93.7% and parameters by 7.2%, while achieving better detection performance. Compared with DDLNet, although the number of parameters increases slightly, our FLOPs are only 1.75 times those of DDLNet, and the F1 score improves significantly (71.36% vs. 66.27%). Notably, compared with LGPNet, which has a similar number of parameters (70.99 M), our parameters are reduced by 46.4%, FLOPs decrease by 89.8%, yet the F1 score is higher by 2.23%. This indicates that DSDGMNet, through a carefully designed dual-stream difference modeling and semantic guidance mechanism, achieves optimal detection accuracy at a relatively economical computational cost. However, we also note that there is still room to optimize the model’s parameter count. Future work will focus on lightweight design to further reduce model complexity while maintaining detection accuracy, thereby better meeting practical deployment needs.

## 4. Conclusions

This paper proposes the DSDGMNet network for detecting mangrove forest changes in tidal disturbance scenarios, focusing on addressing two critical challenges in detecting mangrove distribution changes under varying tidal heights: First, tidal-induced water coverage variations cause significant visual differences between Bi-Temporal images, complicating the identification of genuine changes; second, mangrove distribution changes exhibit high inconsistency at both spatial scales and boundary levels, hindering precise localization. To address these challenges, the network employs a dual-stream difference-driven Bi-Temporal Feature Fusion mechanism to effectively model the differential relationships between Bi-Temporal features. This approach preserves the stable structure of mangroves while enhancing responsiveness to genuine distribution changes. Concurrently, a semantically guided multiscale feature fusion strategy is introduced, enabling adaptive alignment of features across different scales within change areas. This ensures both overall distribution identification and detailed boundary delineation.

Extensive experiments on the self-built GBCNR mangrove change detection dataset demonstrate that the proposed method effectively distinguishes tidal disturbances from mangrove distribution changes in complex intertidal environments, achieving 71.36% F1 and 84.57% recall. We acknowledge that the GBCNR dataset is relatively small due to the high cost of pixel-level annotation in complex mangrove ecosystems, which may limit the statistical power of the evaluation. Nevertheless, validation on public datasets such as WHU-CD (91.38% F1) and LEVIR-CD (90.68% F1) demonstrates the method’s generalization capabilities across diverse change detection scenarios. The model still has room for improvement in parameter scale and computational efficiency. Future research will focus on structural simplification and efficient inference to explore lightweight change detection solutions better suited for large-scale, long-term mangrove monitoring, as well as expanding the dataset to cover more diverse tidal conditions.

## Figures and Tables

**Table 1:** Summary of the proposed architecture.

Module	Input	Output	Output Channels	Output Resolution
ResNet18	$T_{1},T_{2}$	$F_{1}^{i},F_{2}^{i},i=1\ldots 4$,	{64, 128, 256, 512}	{1/4, 1/8, 1/16, 1/32}
DSCF	$F_{1}^{i},F_{2}^{i}$	$X_{i},i=1\ldots 4$	{64, 128, 256, 512}	{1/4, 1/8, 1/16, 1/32}
DFG	$X_{3},X_{4}$	$F_{DFG}$	1	1/32
FR	$F_{out}^{i}$, i=1…4	$Y_{i}$,i=1…4	{64, 128, 256, 512}	{1/4, 1/8, 1/16, 1/32}
DFGF	$X_{i}$, $F_{DFG}$, $Y_{i+1},i=1\ldots 3$	$F_{out}^{i}$	{64, 128, 256, 512}	{1/4, 1/8, 1/16, 1/32}
CAM	$Y_{i}$, i=1…4	output	2	1

**Table 2:** Comparison with the SRC-Net.

Aspect	SRC-Net	Our DSCF
Core idea	Probabilistic mode inference	Explicit difference-guided attention
Fusion strategy	Patch-based multi-head fusion	Dual-branch (difference + connection)
Key difference	Implicit change modeling	Explicit difference as attention prior

**Table 3:** Comparison with the MSFCTNet.

Aspect	MSFCTNet	Our DGMD
Core idea	Full-scale aggregation + gated attention	Progressive decoding + semantic guidance
Guidance	SE-based channel reweighting (implicit)	Explicit semantic map from $X_{3}$, $X_{4}$
Fusion order	All scales → global processing	Layer-by-layer progressive

**Table 4:** The quality control metrics and standards.

Quality Aspect	Metric	Standard
Inter-annotator agreement	Cohen’s Kappa	>0.85
Annotation precision	Mislabeling rate (sampling)	≤2%
Temporal consistency	SSIM	>0.85

**Table 5:** Training time on different datasets.

Dataset	Time per Epoch (min)	Total Training Time (200 Epochs) (h)
GBCNR	0.58	1.94
WHU-CD	2.00	6.67
LEVIR-CD	2.34	7.80
SYSU-CD	3.94	13.13
CLCD	0.29	0.96

**Table 6:** Ablation studies performed on five datasets (%).

Dataset	Methods	Pre	Rec	F1	IoU
GBCNR	Baseline	58.43	81.38	68.02	51.54
	Baseline + DSCF	**62.35**	78.11	69.34	53.07
	DSDGMNet (without DFG)	60.68	83.67	70.35	54.26
	DSDGMNet	61.71	**84.57**	**71.36**	**55.47**
LEVIR-CD	Baseline	**91** **.68**	88.08	90.42	82.09
	Baseline + DSCF	91.53	89.51	90.51	82.67
	DSDGMNet (without DFG)	91.28	89.94	90.61	82.83
	DSDGMNet	91.36	**90.01**	**90.** **68**	**82.95**
WHU	Baseline	86.08	89.84	89.72	78.44
	Baseline + DSCF	91.37	89.99	90.67	82.94
	DSDGMNet (without DFG)	**91.81**	89.75	90.77	83.10
	DSDGMNet	90.92	**91.85**	**91.38**	**84.13**
SYSU	Baseline	80.70	**82.86**	81.76	69.15
	Baseline + DSCF	**86** **.95**	77.59	82.00	69.50
	DSDGMNet (without DFG)	82.99	81.41	82.19	69.77
	DSDGMNet	83.75	79.23	**82.43**	**70.11**
CLCD	Baseline	76.04	71.19	73.54	58.15
	Baseline + DSCF	74.12	75.02	74.57	59.45
	DSDGMNet (without DFG)	73.80	**75.84**	74.81	59.76
	DSDGMNet	**78** **.51**	74.65	**75.31**	**60.39**

The highest score is marked in bold.

**Table 7:** Ablation study on loss functions on the GBCNR dataset (%).

Loss Configuration	Pre	Rec	F1	IoU
Focal only	63.72	76.06	69.35	53.08
Dice only	**63.74**	77.35	69.89	53.71
Combined	61.71	**84.57**	**71.36**	**55.47**

The highest score is marked in bold.

**Table 8:** Effect of different backbone architectures on the GBCNR dataset (%).

Backbone	Pre	Rec	F1	IoU	FLOPs (G)	Params (M)
ResNet-18 (ours)	61.71	84.57	**71.36**	**55.47**	12.84	38.09
ResNet-34	**62.46**	76.64	68.83	52.48	17.68	48.21
ResNet-50	59.16	**81.89**	68.69	52.31	19.40	51.82

The highest score is marked in bold.

**Table 9:** Comparison results on GBCNR dataset (%).

Method	Pre	Rec	F1	IoU
FC-EF [10]	56.97	63.12	59.89	42.74
FC-Sima-diff [10]	61.40	57.05	59.14	41.99
FC-Siam-conc [10]	59.79	54.99	57.29	40.14
LGPNet [34]	61.13	79.54	69.13	52.82
SNUNet [35]	61.63	78.03	68.87	52.52
ChangeFormer [19]	61.09	72.69	66.39	49.69
USSFCNet [36]	59.79	72.20	65.41	48.60
DDLNet [37]	55.89	81.38	66.27	49.55
OURS	**61.71**	**84.57**	**71.36**	**55.47**

The highest score is marked in bold.

**Table 10:** Comparison results on LEVIR-CD dataset (%).

Method	Pre	Rec	F1	IoU
FC-EF [10]	88.53	86.83	87.67	78.05
FC-Sima-diff [10]	**94.02**	82.93	88.13	78.77
FC-Siam-conc [10]	83.81	**91.00**	87.26	77.39
LGPNet [34]	93.07	85.95	89.37	80.78
SNUNet [35]	90.71	89.40	90.05	81.90
ChangeFormer [19]	91.58	89.29	90.42	82.52
USSFCNet [36]	87.66	90.42	89.02	80.21
DDLNet [37]	90.93	89.72	90.32	82.35
OURS	91.36	90.01	**90.68**	**82.95**

The highest score is marked in bold.

**Table 11:** Comparison results on WHU dataset (%).

Method	Pre	Rec	F1	IoU
FC-EF [10]	80.87	75.43	78.05	64.01
FC-Sima-diff [10]	84.73	87.31	86.00	75.44
FC-Siam-conc [10]	78.86	78.64	78.75	64.95
LGPNet [34]	79.87	85.21	82.45	70.15
SNUNet [35]	88.46	83.10	85.70	74.97
ChangeFormer [19]	**92.89**	85.60	88.82	79.89
USSFCNet [36]	91.49	87.28	89.33	80.72
DDLNet [37]	90.03	89.66	89.85	81.56
OURS	90.92	**91.85**	**91.38**	**84.13**

The highest score is marked in bold.

**Table 12:** Comparison results on SYSU-CD dataset (%).

Method	Pre	Rec	F1	IoU
FC-EF [10]	78.26	76.30	77.27	62.96
FC-Sima-diff [10]	83.04	79.11	81.03	68.11
FC-Siam-conc [10]	74.32	75.84	75.07	60.09
LGPNet [34]	79.04	75.69	77.33	63.04
SNUNet [35]	81.76	74.73	78.09	64.06
ChangeFormer [19]	80.71	76.22	78.40	64.47
USSFCNet [36]	82.52	**79.80**	81.14	68.26
DDLNet [37]	84.22	78.80	81.42	68.66
OURS	**85.89**	79.23	**82.43**	**70.11**

The highest score is marked in bold.

**Table 13:** Comparison results on CLCD dataset (%).

Method	Pre	Rec	F1	IoU
FC-EF [10]	70.82	62.37	66.32	49.62
FC-Sima-diff [10]	71.70	47.60	57.22	40.07
FC-Siam-conc [10]	61.42	62.75	62.08	45.01
LGPNet [34]	63.03	64.27	63.64	46.68
SNUNet [35]	69.56	62.16	65.65	48.87
ChangeFormer [19]	60.84	65.04	66.68	50.01
USSFCNet [36]	69.72	61.18	65.17	48.33
DDLNet [37]	**78.10**	71.20	74.49	59.35
OURS	75.98	**74.65**	**75.31**	**60.39**

The highest score is marked in bold.

**Table 14:** Statistical evaluation of the proposed method (%).

Dataset	Pre	Rec	F1	IoU
GBCNR	61.14 ± 0.98	82.26 ± 1.68	70.14 ± 0.74	54.01 ± 0.87
WHU	91.48 ± 0.48	90.89 ± 1.06	91.18 ± 0.35	83.79 ± 0.60

**Table 15:** Model efficiency comparison table.

Method	FLOPs (G)	Params (M)
FC-EF [11]	3.58	1.35
FC-Sima-diff [11]	4.73	1.35
FC-Siam-conc [11]	5.33	1.55
LGPNet [13]	125.80	70.99
SNUNet [12]	54.83	12.04
ChangeFormer [16]	202.78	41.03
USSFCNet [17]	4.86	1.5
DDLNet [14]	7.35	12.67
OURS	12.84	38.09

Params (M) denotes the number of model parameters in millions. FLOPs (G) denotes the floating point operations in billions (Giga). All values are computed with 256 × 256 input size.

## Data Availability

The GBCNR dataset and the code for this study are available from the corresponding author upon reasonable request. The implementation code and trained models for this study are available from the corresponding author on reasonable request.

## References

[1] Friess D.A. (2016). Mangrove Forests. Curr. Biol..

[2] Wang L., Jia M., Yin D., Tian J. (2019). A Review of Remote Sensing for Mangrove Forests: 1956–2018. Remote Sens. Environ..

[3] Yan J., Cheng Y., Wang Q., Liu L., Zhang W., Jin B. (2024). Transformer and Graph Convolution-Based Unsupervised Detection of Machine Anomalous Sound Under Domain Shifts. IEEE Trans. Emerg. Top. Comput. Intell..

[4] Yan J., Cheng Y., Zhang F., Li M., Zhou N., Jin B., Wang H., Yang H., Zhang W. (2025). Research on Multimodal Techniques for Arc Detection in Railway Systems with Limited Data. Struct. Health Monit..

[5] Yan J., Cheng Y., Zhang F., Zhou N., Wang H., Jin B., Wang M., Zhang W. (2025). Multimodal Imitation Learning for Arc Detection in Complex Railway Environments. IEEE Trans. Instrum. Meas..

[6] Pan Y., Xu X., Long J., Lin H. (2022). Change Detection of Wetland Restoration in China’s Sanjiang National Nature Reserve Using STANet Method Based on GF-1 and GF-6 Images. Ecol. Indic..

[7] Pan Y., Lin H., Zang Z., Long J., Zhang M., Xu X., Jiang W. (2023). A New Change Detection Method for Wetlands Based on Bi-Temporal Semantic Reasoning UNet++ in Dongting Lake, China. Ecol. Indic..

[8] Qian S., Xue Z., Jia M., Chen Y., Su H. (2024). Temporal-Spectral-Semantic-Aware Convolutional Transformer Network for Multi-Class Tidal Wetland Change Detection in Greater Bay Area. ISPRS J. Photogramm. Remote Sens..

[9] Luo J., Li J., Chu X., Yang S., Tao L., Shi Q. (2025). BTCDNet: Bayesian Tile Attention Network for Hyperspectral Image Change Detection. IEEE Geosci. Remote Sens. Lett..

[10] Daudt R.C., Saux B.L., Boulch A., Gousseau Y. (2018). Urban Change Detection for Multispectral Earth Observation Using Convolutional Neural Networks. arXiv.

[11] Chen P., Zhang B., Hong D., Chen Z., Yang X., Li B. (2022). FCCDN: Feature Constraint Network for VHR Image Change Detection. ISPRS J. Photogramm. Remote Sens..

[12] Zhou H., Song M., Sun K. (2022). A Full-Scale Feature Fusion Siamese Network for Remote Sensing Change Detection. Electronics.

[13] Zhao S., Zhang X., Xiao P., He G. (2023). Exchanging Dual Encoder-Decoder: A New Strategy for Change Detection with Semantic Guidance and Spatial Localization. IEEE Trans. Geosci. Remote Sens..

[14] Deng J., Gong G., Zhou G., Yan M., Li L. (2024). A Gated Feature Fusion Network With Meta-Encoder and Self-Calibrating Cross Module for Building Change Detection in Remote Sensing Images. IEEE Trans. Geosci. Remote Sens..

[15] Chen H., Xu X., Pu F. (2024). SRC-Net: Bi-Temporal Spatial Relationship Concerned Network for Change Detection. IEEE J. Sel. Top. Appl. Earth Obs. Remote Sens..

[16] Zhang G., Li Z., Tang C., Li J., Hu X. (2025). CEDNet: A Cascade Encoder–Decoder Network for Dense Prediction. Pattern Recognit..

[17] Quan Y., Zhang D., Zhang L., Tang J. (2023). Centralized Feature Pyramid for Object Detection. IEEE Trans. Image Process..

[18] Huang Z., Huang A., Hu X., Hu C., Xu J., Zhou S. (2023). Scale-Adaptive Feature Aggregation for Efficient Space-Time Video Super-Resolution. Proceedings of the IEEE/CVF Winter Conference on Applications of Computer Vision.

[19] Bandara W.G.C., Patel V.M. (2022). A Transformer-Based Siamese Network for Change Detection. IGARSS 2022—2022 IEEE International Geoscience and Remote Sensing Symposium.

[20] Zhang C., Zhang Y., Lin H. (2023). Multi-Scale Feature Interaction Network for Remote Sensing Change Detection. Remote Sens..

[21] Wang L., Li Y., Zhang M., Shen X., Peng W., Shi W. (2023). MSFF-CDNet: A Multiscale Feature Fusion Change Detection Network for Bi-Temporal High-Resolution Remote Sensing Image. IEEE Geosci. Remote Sens. Lett..

[22] Le W., Huang L., Tang B.-H., Tian Q., Wang M. (2024). ACMFNet: Asymmetric Convolutional Feature Enhancement and Multiscale Fusion Network for Change Detection. IEEE J. Sel. Top. Appl. Earth Obs. Remote Sens..

[23] Lu W., Yang X., Chen S.-B. (2025). LWGANet: Addressing Spatial and Channel Redundancy in Remote Sensing Visual Tasks with Light-Weight Grouped Attention. arXiv.

[24] Ma X., Yang J., Hong T., Ma M., Zhao Z., Feng T., Zhang W. (2023). STNet: Spatial and Temporal Feature Fusion Network for Change Detection in Remote Sensing Images. 2023 IEEE International Conference on Multimedia and Expo (ICME).

[25] Hu J., Shen L., Albanie S., Sun G., Wu E. (2020). Squeeze-and-Excitation Networks. IEEE Trans. Pattern Anal. Mach. Intell..

[26] Narayanan M. (2023). SENetV2: Aggregated Dense Layer for Channelwise and Global Representations. arXiv.

[27] Finder S.E., Amoyal R., Treister E., Freifeld O. (2024). Wavelet Convolutions for Large Receptive Fields. European Conference on Computer Vision.

[28] Jiang M., Chen Y., Dong Z., Liu X., Zhang X., Zhang H. (2024). Multiscale Fusion CNN-Transformer Network for High-Resolution Remote Sensing Image Change Detection. IEEE J. Sel. Top. Appl. Earth Obs. Remote Sens..

[29] Chen H., Shi Z. (2020). A Spatial-Temporal Attention-Based Method and a New Dataset for Remote Sensing Image Change Detection. Remote Sens..

[30] Ji S., Wei S., Lu M. (2019). Fully Convolutional Networks for Multisource Building Extraction From an Open Aerial and Satellite Imagery Data Set. IEEE Trans. Geosci. Remote Sens..

[31] Liu M., Chai Z., Deng H., Liu R. (2022). A CNN-Transformer Network With Multiscale Context Aggregation for Fine-Grained Cropland Change Detection. IEEE J. Sel. Top. Appl. Earth Obs. Remote Sens..

[32] Shi Q., Liu M., Li S., Liu X., Wang F., Zhang L. (2022). A Deeply Supervised Attention Metric-Based Network and an Open Aerial Image Dataset for Remote Sensing Change Detection. IEEE Trans. Geosci. Remote Sens..

[33] Lowe D.G. (2004). Distinctive Image Features from Scale-Invariant Keypoints. Int. J. Comput. Vis..

[34] Liu T., Gong M., Lu D., Zhang Q., Zheng H., Jiang F., Zhang M. (2022). Building Change Detection for VHR Remote Sensing Images via Local–Global Pyramid Network and Cross-Task Transfer Learning Strategy. IEEE Trans. Geosci. Remote Sens..

[35] Fang S., Li K., Shao J., Li Z. (2022). SNUNet-CD: A Densely Connected Siamese Network for Change Detection of VHR Images. IEEE Geosci. Remote Sens. Lett..

[36] Lei T., Geng X., Ning H., Lv Z., Gong M., Jin Y., Nandi A.K. (2023). Ultralightweight Spatial–Spectral Feature Cooperation Network for Change Detection in Remote Sensing Images. IEEE Trans. Geosci. Remote Sens..

[37] Ma X., Yang J., Che R., Zhang H., Zhang W. (2024). DDLNet: Boosting Remote Sensing Change Detection with Dual-Domain Learning. 2024 IEEE International Conference on Multimedia and Expo (ICME).

